# GROWING STATE ACTION THROUGH A COMMUNITY OF PRACTICE

**DOI:** 10.1093/geroni/igae098.1559

**Published:** 2024-12-31

**Authors:** Amanda Cheney, John Shean, Erin Bouldin, Meghan Fadel

**Affiliations:** University of Utah, Orem, Utah, United States; Alzheimer’s Association, Chicago, Illinois, United States; University of Utah, Salt Lake City, Utah, United States; Alzheimer’s Association, Chicago, Illinois, United States

## Abstract

Building a community of practice is a key feature of the Data for Action Project. Working alongside a cohort of peers from other states, teams conduct data analysis related to brain health, dementia, and/or caregiving to produce tools for strategic dissemination of resulting data. Arkansas, Connecticut, Illinois, and New Jersey all participated in, and made important contributions to, the pilot cohort. State projects included analysis and dissemination of data on risk factors, dementia service use, and family caregivers. In several states, teams produced estimates among specific sub-populations or regions in the states to identify at-risk populations. Participating state teams also identified ways the data would most help their efforts, including public health planning, budgeting decisions, and public awareness and information efforts. Having data analysts and representatives from the offices in which data were housed (e.g., BRFSS coordinator) were critical to success, as was including diverse perspectives (e.g., aging, disability, and chronic disease) in developing public-facing products and strategies for dissemination, encouraging collaboration, and maintaining future sustainability of the project. Participants in the program reported increased knowledge of the BRFSS optional modules and of the use of data for health improvement planning. 85% of participants agreed that the program positively impacted their health department’s ability to collaborate on integrating brain health and caregiving into public health planning efforts. The second Data for Action cohort launched in 2024, with ongoing efforts to advance brain health in new states.

